# What do we really fear? The epidemiological characteristics of Ebola and our preparedness

**DOI:** 10.4178/epih/e2014014

**Published:** 2014-08-18

**Authors:** Moran Ki

**Affiliations:** Department of Cancer Control and Policy, Graduated School of Cancer Science and Policy, National Cancer Center, Goyang, Korea

**Keywords:** Ebola virus disease, Fatality rate, Outbreak, West Africa, Risk communication

## Abstract

Ebola virus disease (hereafter Ebola) has a high fatality rate; currently lacks a treatment or vaccine with proven safety and efficacy, and thus many people fear this infection. As of August 13, 2014, 2,127 patients across four West African countries have been infected with the Ebola virus over the past nine months. Among these patients, approximately 1 in 2 has subsequently died from the disease. In response, the World Health Organization has declared the Ebola outbreak in West Africa to be a Public Health Emergency of International Concern. However, Ebola is only transmitted by patients who already present symptoms of the disease, and infection only occurs upon direct contact with the blood or body fluids of an Ebola patient. Consequently, transmission of the outbreak can be contained through careful monitoring for fever among persons who have visited, or come into contact with persons from, the site of the outbreak. Thus, patients suspected of presenting symptoms characteristic of Ebola should be quarantined. To date, South Korea is not equipped with the special containment clinical units and biosafety level 4 facilities required to contain the outbreak of a fatal virus disease, such as Ebola. Therefore, it is necessary for South Korea to make strategies to the outbreak by using present facilities as quickly as possible. It is also imperative that the government establish suitable communication with its citizens to prevent the spread of uninformed fear and anxiety regarding the Ebola outbreak.

The current Ebola epidemic has garnered wide media attention throughout the world. As a result, many people fear that the disease, which is generally limited to the African continent, may cause an outbreak in their local community at any given moment.

The present paper will examine the epidemiological characteristics of Ebola, our level of preparedness, and discuss what we fear.

Ebola is a viral disease. Although it has previously been referred to as “Ebola hemorrhagic fever,” some Ebola patients did not present hemorrhage, and thus, it is now referred to as Ebola virus disease. The first known Ebola patient was a 44-year old man who had managed the construction of a school in northern Zaire (currently the Democratic Republic of Congo, DRC). On August 26, 1976, the patient presented at a hospital with a high fever. He received an injection of chloroquine for presumptive malaria and had a clinical remission of his symptoms the next four days. On the sixth day, the patient had a fever of 39.2°C and began to hemorrhage. On September 8 (the 14th day), the patient died with severe hemorrhage. For the following months, until late-October, there was an outbreak of Ebola, with 280 of the 318 patients subsequently dying from the disease [1].

Unfortunately, in the hospital that treated the first case of Ebola, all 17 hospital employees were exposed to the patient, resulting in 13 being infected, and 11 dying. When the pathway of infection for this outbreak was examined, 85 of the 318 patients (26.7%) were found to have received an injection at their respective hospitals. There were no survivors among the patients who were infected through injections. At the time, syringes and needles were sometimes rinsed between patients in a pan of warm water. At the end of the day they were sometimes boiled [1].

When this outbreak was investigated to determine the source of the infection, no particular behavior of interest was presented by the first case prior to infection. Although the patient purchased, cooked, and ate bushmeat (antelopes), other members of his party who had touched and eaten the same bushmeat had not contracted Ebola. During the search through the hospital’s records from January 1974 to the beginning of the epidemic, only one case resembled Ebola. This case was an adult man who was hospitalized on August 20, 1976, with “epistaxis and diarrhea.” The person left against medical advice on August 30, and could not be found, despite an active search during the investigations [1]. It is possible that the first Ebola case was injected using a contaminated syringe or needle that had been used to treat the patient with “epistaxis and diarrhea.”

Since then, Ebola outbreaks have occurred from time to time in Africa over the past 38 years. In this time, there have been 19 additional outbreaks, primarily in central African countries including the DRC (1977, 1995, 2007, 2008, and 2012), Sudan (1976, 1979, and 2004), Gabon (1994, 1996, 2001, and 2002), Uganda (2000, 2007, and 2012), and the Congo (2001-2002, 2003, and two in 2005). There have been 2,403 patients affected during these 19 outbreaks, and 1,594 patients have died from Ebola (66.3%). Although the fatality rate varies depending on the outbreak, country, virus, and period, the most recent outbreak in the DRC during 2012 had a fatality rate of 46.8%, which is a substantially lower than the initial Ebola outbreak (Figure 1).

The current Ebola epidemic is unique from the initial outbreak in West Africa, as it has broken the pattern of outbreaks typically occurring in Central Africa. Beginning in Guinea in December 2013, the present outbreak spread to Sierra Leone, Liberia, and Nigeria, and is now the largest outbreak in history. By August 13, 2014, there were 2,127 cases (suspected and confirmed diagnoses) and 1,145 deaths, representing a 54% fatality rate. By country, Guinea experienced 519 cases, Sierra Leone 810 cases, Liberia 786 cases, and Nigeria 12 cases, with fatality rates of 73.2%, 43.0%, 52.5%, and 33.3%, respectively (Table 1). The first case in Guinea was a 2-year-old male child who began to present symptoms on December 2, 2013, and died six days later presenting symptoms typical of Ebola infection. Later, the family of the child (a 3-year old sister, mother, and grandmother) and the medical team (nurse and village midwife) also died from Ebola. Subsequently, 14 people, including the family of the midwife, funeral attendees, and health worker from the same local hospital also died. The disease then spread through the family of the health worker [2] Unfortunately, Ebola was not suspected, reported, or confirmed until after mid-March 2014. By this time, the peak of the first outbreak wave had already passed, and in May 2014, the second outbreak wave began, spreading nearby regions, including Sierra Leone and Liberia.

Although the transmission of Ebola infection appears high, this is not true. According to a study that examined the outbreaks in DRC (1995) and Uganda (2000), the basic reproductive number (the average number of secondary infections generated by one primary case in an entirely susceptible population) of Ebola was shown to be 2.7 [3], and has also been ranging from 1.34 to 3.65 [4,5]. However, if the necessary interventions are performed, then the effective reproduction number decreases dramatically to 0.3-0.4. This reduction is possible, as transmission occurs primarily through hospitals and funerals, where it can easily be controlled [3]. In disease epidemiology, if the reproductive number is less than 1, this means a patient with the disease cannot transmit the disease to another individual during the infection period. Thus, the outbreak cannot be sustained, and eventually ends.

Many people have questions regarding the source of Ebola infections, although there are currently no clear indicators regarding their source. Fruit bats of the Pteropodidae family are considered the natural host of the Ebola virus, which is also thought to transmit through monkeys, gorillas, and chimpanzees. However, the initial human-to-human transmission must occur through contact with the body fluids of an infected patient. Thus, Ebola is not airborne, as is the case with influenza, nor is it food or waterborne, as is the case with other diarrheal diseases (cholera, dysentery, or typhoid). Furthermore, the Ebola virus does not infect other individuals during the incubation period (the period between the initial infection and the onset of symptoms), which for the Ebola virus can be 2-21 days (generally 8-9 days). As a result, if a person who has been in contact with an Ebola patient, or a patient suspected of Ebola presented fever, immediate quarantine, treatment, and management by the hospital can halt the spread of the outbreak. However, even after recovery, the virus may still found in the body fluids of the patient for an extended period. In one report, the Ebola virus was detected in the semen of the patient three months after recovery [7]. Thus, it is important that the patient only can be released from isolation after confirming that the Ebola virus is no longer present. Although there are currently no established treatments or vaccines of Ebola available worldwide, symptomatic therapy and strict quarantine are adequate to prevent transmission of an Ebola.

Despite these solutions, it is curious that the current outbreak in West Africa has persisted and amplified over the past nine months. The first reason is clearly that an Ebola outbreak was initially not suspected, which allowed the outbreak to amplify and spread to other regions. The second reason is that an inadequate medical system actually amplified the Ebola outbreak, rather than contained it. The third reason is that the site of the initial outbreak bordered three countries, unlike previous outbreaks that were in distant rural areas. As such, the outbreak spread to the capital of the three countries, accelerating the spread of the outbreak. The final reason that the current outbreak has not yet been mitigated is that during traditional funeral proceedings in Africa mourners often physically touch the body of the deceased. According to the recent report released by the World Health Organization (WHO), traditional funeral proceedings have led to infections among many relatives and friends of the deceased, and 60% of Ebola infections in Guinea have been associated with such funeral proceedings [8].

On August 8, 2014, WHO declared the present West Africa Ebola outbreak as a Public Health Emergency of International Concern. Consequently, public health partnerships between the involved countries are expected to be expanded, and the national response systems will be in effect. Each country must strengthen their own preparedness and response system to prevent an Ebola outbreak, and support these four countries in controlling this epidemic. It is also important to investigate the epidemiologic characteristics (such as incubation period, latent period, transmission period and pathway, pathogenicity, virulence, fatality, basic reproduction number, etc.) of Ebola, including public health data (such as health behaviors, the knowledge on Ebola, related cultural characteristics, etc.), which can provide basic data to prevent future outbreaks of Ebola.

What is the level of preparedness in South Korea?

What if an Ebola patient entered South Korea and the patient visited a hospital due to a fever?

First, as this hypothetical patient did not present symptoms during the immigration process, the possibility of the patient infecting the surrounding population is nearly zero. Although the patient would likely encounter many different people while undergoing testing in the hospital, the Ebola virus is not airborne and is only transmitted through contact with body fluids. As such, the possibility of infection of the medical and administrative team is very low. However, what if the patient presented severe symptoms and visited the hospital while hemorrhaging? At this point, there is a significant risk of infection for family and medical professionals that came into contact with the patient during patient care and transport. As well, there is a risk of medical professionals in the emergency room becoming infected, as they would be exposed to the body fluids of the patient while attempting to treat the hemorrhage. Although the possibility of this hypothetical situation occurring is extremely low, we should be thoroughly prepared for such an eventuality.

Another question is: are there any specialized quarantine hospitals equipped to treat Ebola patients in South Korea? Although there are 17 government-designated hospitals with quarantine units, these units were constructed for airborne infectious diseases like influenza. As such, currently no hospitals in South Korea are designed to provide one-stop testing and treatment for diseases like Ebola that are transmitted through body fluids.

Finally, if the Ebola virus is separated from the host, does South Korea have facilities that can safely handle the strains? The Ebola virus belongs to the highest biosafety level designation (biosafety level 4, BL4). BL4 facility is a standalone building that contains designated shower rooms. Furthermore, no one may enter a BL4 facility without a positive-pressure personal protection suit equipped with a segregated air supply. Unfortunately, there are currently no BL4 facilities in South Korea. Consequently, a lower level (BL3) facility must be used to handle Ebola virus in South Korea currently. The BL3 facility that is designated to handle Ebola virus should be prepared to maximize the safety of the personnel handling the Ebola samples. Furthermore, the government-designated quarantine units should be prepared as soon as possible to include special containment units with specimen isolation capacity. Finally, a channel is needed to provide open, real-time communication with citizens regarding all situations and responses to an Ebola outbreak. Proper risk communication and an understanding of Ebola will be the most important steps to avoid the unnecessary anxiety, fear, and excessive reaction that is linked to our greatest fear: the fear of the unknown.

## Figures and Tables

**Figure 1.**
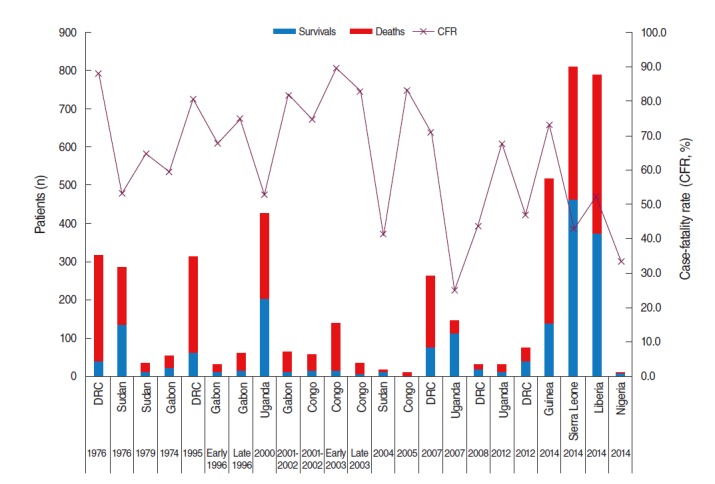
Survival and fatality rates associated with Ebola outbreaks (as of Aug 13. 2014). DRC, Democratic Republic of Congo. From World Health Organization. Ebola virus disease update-west Africa [6].

**Table 1.** Confirmed outbreaks of Ebola virus disease (excluding isolated cases)

Location	Virus	Year	Cases	Death	CFR (%)
DRC	Zaire	1976	318	280	88.1
Sudan	Sudan	1976	284	151	53.2
Sudan	Sudan	1979	34	22	64.7
Gabon	Zaire	1994	52	31	59.6
DRC	Zaire	1995	315	254	80.6
Gabon	Zaire	Early 1996	31	21	67.7
Gabon	Zaire	Late 1996	60	45	75.0
Uganda	Sudan	2000	425	224	52.7
Gabon	Zaire	2001-2002	65	53	81.5
Congo	Zaire	2001-2002	59	44	74.6
Congo	Zaire	Early 2003	143	128	89.5
Congo	Zaire	Late 2003	35	29	82.9
Sudan	Sudan	2004	17	7	41.2
Congo	Zaire	2005	12	10	83.3
DRC	Zaire	2007	264	187	70.8
Uganda	Bundibugyo	2007	149	37	24.8
DRC	Zaire	2008	32	14	43.8
Uganda	Sudan	2012	31	21	67.7
DRC	Bundibugyo	2012	77	36	46.8
Subtotal			2,403	1,594	66.3
Guinea	Zaire	2014	495	367	74.1
Sierra Leone	Zaire	2014	717	298	41.6
Liberia	Zaire	2014	554	294	53.1
Nigeria	Zaire	2014	13	2	15.4
Subtotal			1,779	961	54.0

Data current as of August 13, 2014.

CFR, Case-fatality ratio (deaths/cases×100); DRC, Democratic Republic of Congo (formerly Zaire).

From World Health Organization. Ebola virus disease update-west Africa [6].
