# Supplementary material for: What do we really fear? The epidemiological characteristics of Ebola and our preparedness
Source: Epidemiol Health. 2014 Aug 18;36:e2014014. doi: 10.4178/epih/e2014014 (PMC4153011; doi:10.4178/epih/e2014014)
Supplement: Supplementary file 1 [file epih-36-e2014014-supplemetal data.pdf]

진정 두려운 것은 무엇인가? : 에볼라 역학적 특징과 우리의 대응 방안

기모란

에볼라 바이러스병은 치명률이 높고 효과와 안전성을 모두 검증받은 치료제나 백신이 없어 많은 사람들이 두려워하고 있는 질환이다. 이번에 서아프리카에서 9 개월째 지속된 유행으로 8 월 13 일까지 4 개국에서 2127 명의 환자가 발생하였고 1145 명이 사망하여 2 명중 1 명꼴인 높은 치명률을 보이고 있다. 이에 WHO 에서는 국제보건긴급상황을 선포하였다. 하지만 에볼라 바이러스는 증상이 나타난 이후에 전염되며 환자의 혈액이나 체액에 접촉한 경우에만 감염이 된다. 따라서 유행지역을 방문하였거나 접촉한 사람을 대상으로 발열감시와 함께 환자 격리를 철저히 시행한다면 유행 전파를 막을 수 있다. 한국은 아직 에볼라 바이러스와 같은 질환에 적합한 격리병상이나 실험실 안전 4 등급 시설이 없다. 이에 대한 대비를 빠른 시간에 마련하여 대응하는 것이 필요하며, 국민과의 소통을 통해서 막연한 불안과 두려움을 예방하는 것이 무엇보다 중요하다.

Ebola virus disease, Fatality rate, Outbreak, Africa, Risk communication

에볼라가 전세계 뉴스의 1 면을 장식하고 있다. 많은 사람들이 영화에서나 보던, 멀리 아프리카에서 가끔 유행하는 것으로 알던 질병이 당장 내 주변에서 발생할 것처럼 두려워하고 있다.

이 글에서 에볼라의 역학적 특성과 우리의 대응 수준을 살펴보고 우리가 진정 두려워하는 것이 무엇인지 논의해보고자 한다.

에볼라는 바이러스 질환이다. 전에는 에볼라 출혈열 (Ebola haemorrhagic fever)로 불리웠으나 출혈이 없는 환자들도 있기 때문에 이제는 에볼라 바이러스병(Ebola virus disease, EVD)로 불리고 있다. 첫 에볼라 환자로 알려진 사람은 자이레 (현재의 콩고민주공화국 Democratic Republic of Congo (이후 DRC)) 북쪽 지방에서 학교건축을 관리하던 44 세 남자이다. 그는 1976 년 8 월 26 일 열이 나서 병원에 갔고 말라리아 치료를 받았고 이후 4 일간은 열도 내리고 괜찮다가 6 일째에 다시 39.2 도까지 열이

오르면서 출혈증상이 나타났고 14 일째인 9 월 8 일 과다출혈로 사망했다. 이후 10 월말까지 2 달동안 유행이 발생하여 총 318 명의 환자가 발생하였고 이중에 280 명이 사망하였다. 특히 첫 환자를 치료하였던 병원에서는 병원직원 17 명이 모두 환자와 접촉하였는데 13 명이 질병에 걸렸고 그 중 11 명이 사망했다. 그런데 318 명 환자의 감염 경로를 살펴보면 26.7%인 85 명이 병원에서 주사를 맞았고 그 당시 주사기는 하루에 한번만 가열소독을 하였으며 중간에는 단순히 세척만 하였다고 한다. 또한 감염 경로별로 살펴본 결과 불행히도 이렇게 오염된 주사에 의해 감염된 환자들 중에서 생존자는 없었다.

그럼 첫 환자는 어떻게 감염이 된 것일까? 그 당시 유행 역학조사 결과를 보면 이 환자의 감염 전 행동은 특별한 것이 없었다. 야생동물(영양)을 사다 요리하여 먹었지만 이 동물을 만지고 요리하거나 같이 먹었던 다른 모든 사람들은 발병하지 않았다. 그런데 역학자들이 1976 년 1 월부터 병원의 모든 환자 기록을 살펴본 결과 단 한명의 환자만이 에볼라와 유사한 증상을 보였다. 이 환자는 "코피와 설사"로 입원한 성인 남자 환자로 8 월 20 일부터 30 일까지 입원 치료를 받고 퇴원하였다. 안타깝게도 이 환자에 대한 추적조사는 이루어지지 못하여서 이 환자가 에볼라에 감염되었었는지는 알 수 없다. [1] 다만 같은 기간 입원하였던 첫번째 환자가 이 환자에게 사용되었던 주사를 맞았을 가능성은 충분하다.

이후 38 년동안 아프리카에서는 에볼라 유행이 때때로 발생하였다. 같은 해 수단에서 유행이 있었고(자이레보다 조금 먼저 발생하였다고 한다), 콩고민주공화국(1977, 1995, 2007, 2008, 2012), 수단 (1976, 1979, 2004), 가봉 (1994, 1996, 2001, 2002), 우간다 (2000, 2007, 2012), 콩고 (2001-2002, 2003 년 2 회, 2005) 등으로 주로 중앙아프리카에서 발생하였다. 19 번의 이 유행 동안 총 2403 명의 환자가 발생했고 이중에 66.3%인 1594 명이 사망하였다. 치명률은 유행마다, 국가마다, 시기별로 다르지만 가장 최근 2012 년 콩고민주공화국유행에서는 46.8%로 초기 유행보다는 감소하였다. (Fig 1)

이번 에볼라 유행은 그동안 중앙아프리카에서 주로 발생하던 양상에서 처음으로 서아프리카에서 나타난 유행이다. 2013 년 12 월, 기니에서 처음 시작하여 시에라리온, 라이베리아, 나이지리아로 확산되면서 지금까지 에볼라 유행 중에 가장 큰 규모로 번지고 있다. 2014 년 8 월 13 일까지 환자(의심, 확진 모두 포함) 발생은 총 2127 명, 사망자는 1145 명으로 치명률은 54%이다. 국가별로는 기니 519 명, 시에라리온 810 명, 라이베리아 786 명, 나이지리아 12 명 이고, 치명률은 각각 73.2%, 43.0%, 52.5%,

33.3%이다. (Table 1) 기니의 치명률이 높은 것은 유행 초기에 에볼라를 인지하지 못한 상태에서 치명률이 높았기 때문이다.

기니의 첫환자는 2 세 남자아이로 2013 년 12 월 2 일에 증상이 시작되어 6 일에 사망하였다. 이후 이 아이의 가족(3 세 누나, 엄마, 할머니)과 의료진(간호사, 마을 조산사)이 에볼라로 사망하였고 조산사의 가족, 장례 참석자, 같은 지역병원 보건요원 등 14 명이 사망하였으며, 지역병원 보건요원을 돌보던 가족과 같은 병원의 의료진을 통해서 질병이 증폭 확산되었다. [2]

그런데 에볼라를 의심하여 보고하고 확인이 이루어진 것은 3 월 중순이 넘어서였다. 이때는 이미 첫번째 유행 파도의 정점을 지나고 있던 시점이었다. 이후 5 월부터 2 차 유행파도가 시작되었고 이번에는 인근 지역이었던 시에라리온과 라이베리아에서도 유행 파도가 시작되었다.

에볼라의 감염 전파력은 매우 높은 것으로 보이지만 사실은 그렇지 않다. 콩고민주공화국 유행(1995)과 우간다 유행(2000)을 기초로 한 연구 결과에 의하면 에볼라의 기초재생산수(환자 한명이 완전한 감수성자 집단에서 발생시키는 2 차 감염자의 수) 는 2.7 이었고 [3], 그 범위는 1.34-3.65 로 보고되고 있다. [4, 5]이었다. 그런데 감염관리 조치가 취해진다면 실제재생산수(effective reproduction number)는 0.3-0.4 정도로 급격히 낮아진다고 하였다. 이렇게 낮아질 수 있는 이유는 감염 전파가 병원과 장례절차 등을 통해서 주로 이루어진 것이므로 충분히 막을 수 있기 때문이다. [3] 질병 역학에서 재생산수가 1 보다 낮으면 환자 한명이 감염 가능기간동안 1 명에게도 전파시키지 못하므로 유행은 지속될 수 없어 곧 종식된다.

Table 1. Confirmed outbreaks of Ebola virus disease (excluding isolated cases)

Location	Virus	Year	Cases	Deaths	CFR(%)
DRC	Zaire	1976	318	280	88.1
Sudan	Sudan	1976	284	151	53.2
Sudan	Sudan	1979	34	22	64.7

Gabon	Zaire	1994	52	31	59.6
DRC	Zaire	1995	315	254	80.6
Gabon	Zaire	Early 1996	31	21	67.7
Gabon	Zaire	Late 1996	60	45	75.0
Uganda	Sudan	2000	425	224	52.7
Gabon	Zaire	2001-2002	65	53	81.5
Congo	Zaire	2001-2002	59	44	74.6
Congo	Zaire	Early 2003	143	128	89.5
Congo	Zaire	Late 2003	35	29	82.9
Sudan	Sudan	2004	17	7	41.2
Congo	Zaire	2005	12	10	83.3
DRC	Zaire	2007	264	187	70.8
Uganda	Bundibugyo	2007	149	37	24.8
DRC	Zaire	2008	32	14	43.8
Uganda	Sudan	2012	31	21	67.7
DRC	Bundibugyo	2012	77	36	46.8
Subtotal			2403	1594	66.3
Guinea	Zaire	2014	519	380	73.2
Sierra Leone	Zaire	2014	810	348	43.0
Liberia	Zaire	2014	786	413	52.5

Nigeria	Zaire	2014	12	4	33.3
Subtotal			2127	1145	53.8

* CFR, Case-fatality rate (deaths / cases * 100); DRC, Democratic Republic of Congo (formerly Zaire)

**As of August 13, 2014. [6]

에볼라의 감염원(source of infection)은 무엇일까? 지금까지 유행에서 정확히 밝혀진 적은 없었다. 하지만 아프리카 지역의 과일박쥐 (Fruit bats, Pteropodidae family) 가 에볼라 바이러스의 자연 숙주로 알려져 있다. (그리고 원숭이, 고릴라, 침팬지 등을 통해서 전파된다고 한다. 하지만 최초 환자에서 사람간 전파는 환자의 혈액이나 체액 등과 접촉을 통해서 이루어진다. 따라서 인플루엔자같이 호흡기로 전파된다거나, 다른 설사질환(콜레라, 이질, 장티푸스 등)처럼 음식이나 물로 전파되지 않는다. 또한 무증상 잠복기(환자가 감염된 후 증상이 나타날때까지 기간, 에볼라는 2-21 일) 동안 다른 사람을 감염시키지도 않는다. 따라서 에볼라 환자 접촉자나 의심 환자가 발열 증상을 보이면 즉각 격리하여 병원에서 제대로 치료, 관리한다면 유행이 퍼져나가지 않는다. 하지만 회복 후 환자의 체액에서는 한동안 바이러스가 발견된다고 한다. 한 연구에서는 회복 후 3 개월까지도 환자의 정액에서 바이러스가 발견되었다고 한다. [7] 따라서 에볼라에서 회복된 환자라도 바이러스 배출이 더 이상 없다는 것이 확인된 후에 격리 해제를 하는 것이 중요하다. 아직까지 전세계적으로 효과와 안전성이 입증된 에볼라 치료제나 백신이 없는 상황이지만 대증요법과 함께 전파를 막기위한 철저한 격리 치료를 한다면 충분히 유행을 예방할 수 있을 것이다.

그럼에도 불구하고 아프리카에서 9 개월동안 유행이 지속되어 커진 이유는 무엇일까? 아마도 초기에 에볼라를 의심하지 못하고 유행을 키운 것이 첫번째 원인이 될 것이고 두번째는 의료시스템이 제대로 갖추어져 있지 않아 병원을 통해서 오히려 유행을 확산시킨 것이 될 것이며, 세번째는 이번 유행 발생 지역이 이전 유행과 달리 3 개국의 접경지역이었고, 이후 3 개국의 수도로 전파되면서 유행 속도가 커진 것이 될 것이다. 마지막으로는 아프리카의 전통 장례 방식 (조문객이 시신을 만지는) 때문 이기도 하다. 최근 WHO 의 보고에 의하면 전통적인 방법으로 장례를 치르면서 많은 친지들, 친구들의

감염이 일어나고 있다고 한다. 특히 기니는 환자의 60%가 이러한 장례와 연관되어 있다고 한다. [8]

WHO 는 8 월 8 일을 기해서 이번 서아프리카 에볼라 유행을 '국제적 공중보건 긴급사태(PHEIC: Public Health Emergency of International Concern)'로 선언하였다. 이에 따라 각국의 공중보건 협력 논의가 확대될 예정이고, 국가별 대응 체제가 발효될 예정이다. 각국은 에볼라 환자의 유입을 막기 위한 대비 대응체계 마련과 함께 힘을 합쳐 서아프리카 4 개국을 지원하여 유행을 종식시키도록 노력해야 할 것이다. 또한 에볼라에 대한 좀더 명확한 역학적 특성을 파악하는 것도 중요하다. 다양한 역학적 지표(잠복기, 잠재기와 전파시기와경로, 감염력, 병원성, 치명률, 재생산수등)서아프리카 주민들의 건강행태, 에볼라 바이러스 병에 대한 인식, 관련된 문화적 특성 등을 조사하여 유행 확산의 원인을 구명하는 것이 향후 유행을 방지하는데 중요한 기초자료가 될 것이다.

한국의 대응 수준은 어떠한가?

한국에 에볼라 환자가 입국하였다면? 그리고 그 환자가 열이 나서 병원을 찾아갔다면 어떻게 될까?

우선 입국당시 증상이 발생하지 않았으므로 비행기나 공항 등에서 다른 사람을 감염시킬 확률은 거의 없다. 병원에 가서 검사를 받는 동안 여러 사람을 만났겠지만 호흡기 전파가 아니라 환자의 혈액, 체액 등을 직접 만져야 전파가 되므로 병원의 의료진, 행정 인력 등의 감염 확률도 매우 낮다. 그러나 만약 환자가 증상이 심해져서 출혈이 되는 상태로 병원에 갔다면? 그때는 환자 간호나 이송을 위해서 접촉한 가족이나 의료인 등의 감염 위험이 있다. 또한 응급실에서 환자의 출혈 상태를 처치하려고 애쓰다 혈액이나 체액에 노출된 의료인의 감염 위험성이 있다. 이러한 상황 설정은 매우 가능성이 적지만 발생 가능성을 염두에 두고 대비는 철저히 해야 할 것이다.

그럼 에볼라 환자가 발생했을 때 치료할 전문 격리병원이 있나? 한국에는 국가 지정 격리병상을 운영하는 병원이 17 개 있지만 인플루엔자 같은 호흡기 감염병을 가정하고 만든 것이어서 에볼라처럼 혈액, 체액 등으로 전파되는 경우를 고려하여 모든 검사 시료까지 격리된 곳에서 한번에 다룰 수 있도록 설계된 병원은 아직 없다. 또한 에볼라 바이러스가 분리되었다고 하면 이를 다룰 수 있는 실험실은 있나? 에볼라 바이러스는 가장 높은 단계인 생물안전 4 등급(Bio-safety level 4, BL 4)에 해당된다. BL4 실험실은

별도로 설계된 건물로 샤워실이 필요하고 방호복이 없으면 입실할 수 없다. 또한 별도의 공기튜브가 옷에 연결되어 있어 호흡하도록 되어있다. 한국에는 아직 BL4 실험실은 없다. 따라서 현재 상황에서는 이보다 낮은 단계인 BL3 실험실을 이용하여야 한다. 그렇다면 BL3 를 이용하되 최대한 실험자의 안전을 보장하고 에볼라 바이러스를 다룰 수 있는 준비가 필요할 것이다. 또한 빠른 시간내에 국가지정 격리병상 중에 검체 격리까지 가능한 병상을 준비하는 것이 필요하다. 또한 이러한 모든 상황과 대처에 대하여 국민들에게 실시간으로 솔직히 알리는 소통이 필요하다. 제대로 된 의사소통, 질병에 대한 이해는 불필요한 불안, 두려움, 과잉 반응을 막을 수 있는 가장 중요한 방안이 될 것이다. 왜냐하면 진정 두려운 것은 적을 알지 못하는 데서 오기 때문이다.

1.Breman JG, Piot P, Johnson K, White MK, Mbuyi M, Sureau P, et al. Ebola haemorrhagic fever in Zaire, 1976. Bull World Health Organ 1978;56(2):271-293.
2.Baize S, Pannetier D, Oestereich L, Rieger T, Koivogui L, Magassouba N, et al. Emergence of Zaire Ebola Virus Disease in Guinea - Preliminary Report. N Engl J Med 2014
3.Legrand J, Grais RF, Boelle PY, Valleron AJ, Flahault A. Understanding the dynamics of Ebola epidemics. Epidemiol Infect 2007;135(4):610-621.
4.Chowell G, Hengartner NW, Castillo-Chavez C, Fenimore PW, JM H. The basic reproductive number of Ebola and the effects of public health measures: the cases of Congo and Uganda. Journal of Theoretical Biology 2004;229:119-126.
5.Ferrari MJ, Bjornstad ON, Dobson AP. Estimation and inference of R0 of an infectious pathogen by a removal method. Math Biosci 2005;198(1):14-26.
6.WHO. Ebola virus disease update - west Africa [cited 2014 Aug 17]. Available from: http://www.who.int/csr/don/2014_08_15_ebola/en/.
7.Bausch DG, Towner JS, Dowell SF, Kaducu F, Lukwiya M, Sanchez A, et al. Assessment of the risk of Ebola virus transmission from bodily fluids and fomites. J Infect Dis 2007;196 Suppl 2:S142-147.
8.WHO. Barriers to rapid containment of the Ebola outbreak [cited 2014 Aug 17]. Available from: http://www.who.int/csr/disease/ebola/overview-august-2014/en/.

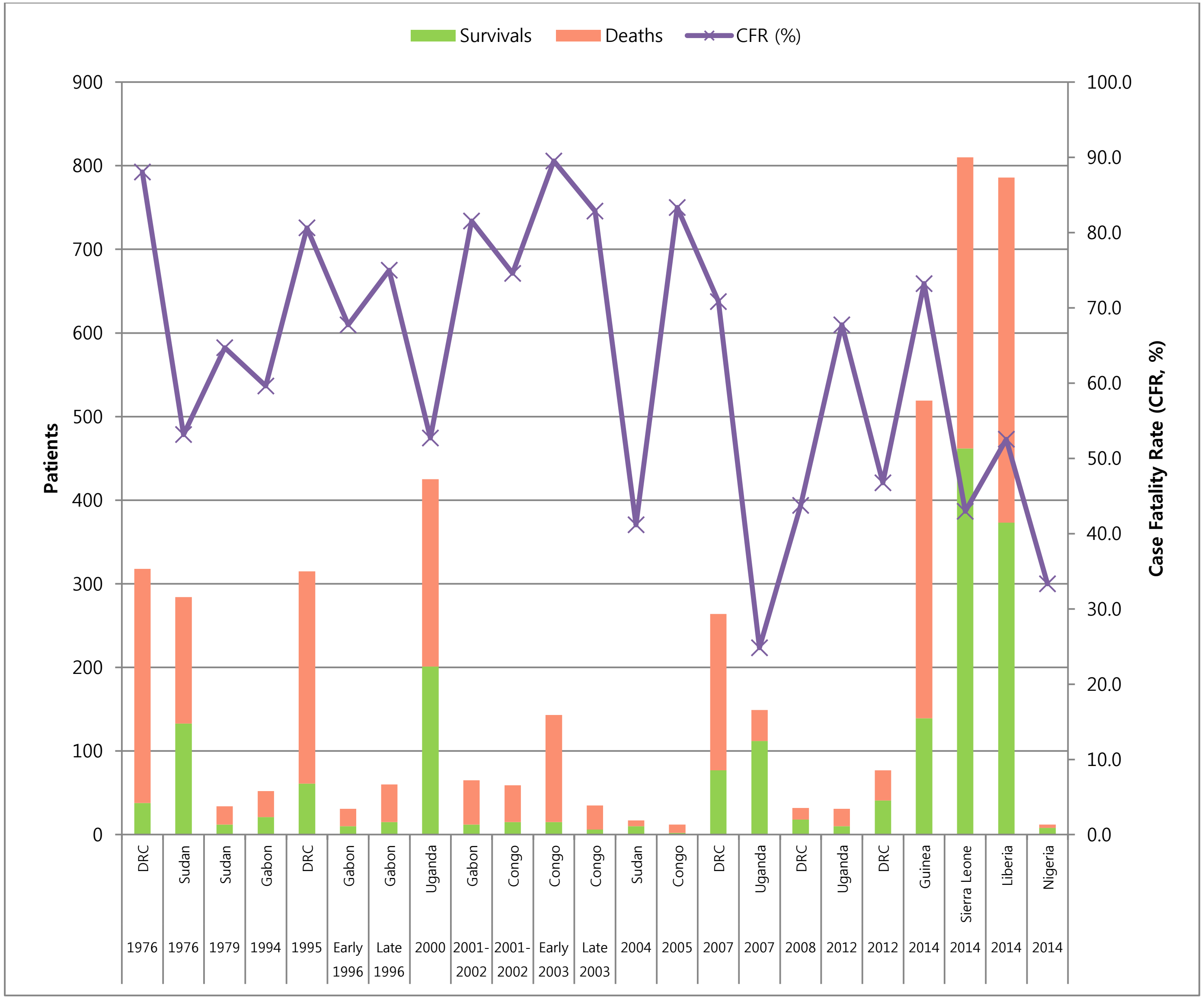
Survivals
Deaths
CFR (%)
Patients
Case Fatality Rate (CFR, %)
900
800
700
600
500
400
300
200
100
0
100.0
90.0
80.0
70.0
60.0
50.0
40.0
30.0
20.0
10.0
0.0
DRC 1976
Sudan 1976
Sudan 1979
Gabon 1994
DRC 1995
Gabon Early 1996
Gabon Late 1996
Uganda 2000
Gabon 2001-2002
Congo 2001-2002
Congo Early 2003
Congo Late 2003
Sudan 2004
Congo 2005
DRC 2007
Uganda 2007
DRC 2008
Uganda 2012
DRC 2012
Guinea 2014
Sierra Leone 2014
Liberia 2014
Nigeria 2014

Figure 1. Survival and fatality rates associated with Ebola outbreaks. (As of Aug 13, 2014)
